# Identification of developing multiple organ failure in sepsis patients with low or moderate SOFA scores

**DOI:** 10.1186/s13054-018-2084-z

**Published:** 2018-06-05

**Authors:** Gunnar Elke, Frank Bloos, Darius Cameron Wilson, Patrick Meybohm, the SepNet Critical Care Trials Group

**Affiliations:** 1 Department of Anaesthesiology and Intensive Care Medicine, University Medical Center Schleswig-Holstein, Campus Kiel, Arnold-Heller-Str. 3 Haus 12, 24105 Kiel, Germany; 2 Deparment of Anesthesiology and Intensive Care Medicine, Jena University Hospital, Am Klinikum 1, 07747 Jena, Germany; 3 Center for Sepsis Control & Care (CSCC), Jena University Hospital, Am Klinikum 1, 07747 Jena, Germany; 4 B·R·A·H·M·S GmbH, Hennigsdorf, Neuendorfstr. 25, 16761 Hennigsdorf, Germany; 5 Department of Anaesthesiology, Intensive Care Medicine and Pain Therapy, University Hospital Frankfurt, Theodor-Stern-Kai 7, 60590 Frankfurt am Main, Germany

An early identification of sepsis patients likely to progress towards multiple organ failure is crucial in order to initiate targeted therapeutic strategies to decrease mortality. Our recent publication highlighted the greater accuracy of mid-regional proadrenomedullin (MR-proADM) compared with conventional biomarkers and clinical scores in predicting 28-day mortality in patients with initially low (≤7 points; *N* = 240) or moderate (8–13 points; *N* = 653) Sepsis-related Organ Failure Assessment (SOFA) scores [1], thus confirming results from smaller investigations [2, 3]. This additional post hoc analysis aimed to further describe the non-surviving patient population of both subgroups and identify those likely to progress towards sepsis-related multiple organ failure.

In our study, patients with low SOFA scores had a lower 28-day mortality rate (*N* = 35; 14.6% vs. *N* = 181; 27.7%) and incidence of septic shock [4] (*N* = 87; 36.7% vs. *N* = 399; 61.5%) compared to those with moderate values. Nevertheless, multiple organ failure was the most common cause of death irrespective of initial SOFA classification (low vs. moderate SOFA: *N* = 16; 45.7% vs. *N* = 79; 43.6%). Patients with low SOFA scores tended to take longer to progress towards multiple organ failure (10 [6–18] vs. 7 [3–11] days) and had an increasing number of dysfunctional organs (identified by organ-specific SOFA scores ≥2) and an increasing overall SOFA score (e.g. diagnosis to day 7: 2 [1–2] vs. 4 [3–5] dysfunctional organs; *P* < 0.01; 6.3 ± 1.3 vs. 10.2 ± 4.7 points; *P* < 0.01).

**Fig. 1:**
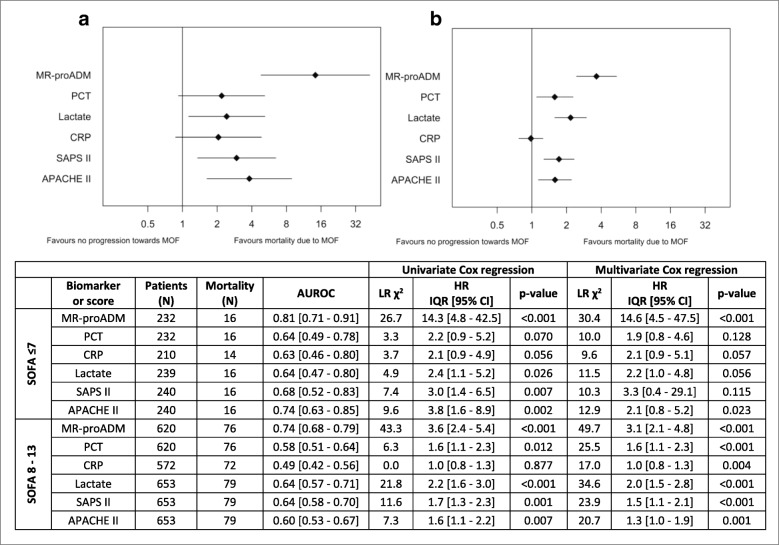
Prediction of sepsis-related multiple organ failure in low (≤7 points) and moderate (8–13 points) SOFA severity patient populations. Cox regression and AUROC analysis for 28-day mortality due to sepsis-related multiple organ failure. Univariate Cox regression was compared for each biomarker and clinical score in the (**a**) low (≤7 points) and (**b**) moderate (8–13 points) SOFA severity subgroups. Multivariate Cox regression analysis was corrected for age and the presence of comorbidities. *Abbreviations*: *APACHE II* Acute Physiology and Chronic Health Evaluation II (score), *AUROC* area under the receiver operating characteristic curve, *CI* confidence interval, *CRP* C-reactive protein, *HR* hazard ratio, *IQR* interquartile range, *LR* likelihood ratio, *MOF* multiple organ failure, *MR-proADM* mid-regional proadrenomedullin, *N* number, *PCT* procalcitonin, *SAPS II* Simplified Acute Physiology Score II, *SOFA* Sepsis-related Organ Failure Assessment

Area under the receiver operating characteristic curve (AUROC) and Cox regression analysis indicated that MR-proADM had the highest accuracy in predicting progression towards sepsis-related multiple organ failure mortality in both groups (Fig. 1). High initial concentrations in non-surviving patients with low or moderate SOFA scores resulted in a high progression rate towards multiple organ failure (*N* = 6; 100.0% and *N* = 25; 52.1%), with similar results found in patients with increasing concentrations over the first 24 h (e.g. moderate SOFA population: *N* = 15; 57.8%). Conversely, mortality in patients with low MR-proADM concentrations was predominantly due to non-sepsis-related causes (*N* = 14; 60.9%), with a low subsequent progression rate towards sepsis-related multiple organ failure in the total patient population with continuously low concentrations over the first 24 h (*N* = 3; 1.4%).

Results suggest that initially high or increasing MR-proADM concentrations may help to identify patients with a high risk of progression towards sepsis-related multiple organ failure. Elevated microcirculation dysfunction and endothelial permeability may therefore play a significant role in driving the development of further organ dysfunction, as described previously [5]. Further studies in larger patient populations are essential to confirm these hypotheses.
